# Prescribing carbonic anhydrase inhibitors to patients with “sulfa” antibiotics allergy: do we dare?

**DOI:** 10.1038/s41433-025-03674-9

**Published:** 2025-02-15

**Authors:** Raquel Quintanilla, Luke van Leeuwen, Arjun Sharma, Ta Chen Chang, Elizabeth Hodapp, John McSoley, Alana Grajewski, Elena Bitrian

**Affiliations:** Bascom Palmer Eye Institute, University of Miami Miller School of Medicine, Miami, FL USA

**Keywords:** Adverse effects, Education

## Abstract

**Objective:**

To evaluate if provider characteristics affect attitude toward carbonic anhydrase inhibitors (CAI) prescription for patients with history of sulfonamide antibiotic (SA) hypersensitivity.

**Methods:**

A survey querying providers’ attitudes toward CAI prescription in hypothetical patients with prior SA hypersensitivity was distributed to four ophthalmology and optometry organizations. Logistic regression was used to assess the relationship between avoiding CAI and profession, specialty, organizational affiliation, and years in practice.

**Results:**

Of 250 respondents, 27% and 52% would avoid topical and oral CAI, respectively, in patients with prior SA rash and/or urticaria. >90% would avoid oral CAI in patients with prior severe SA hypersensitivity. Respondents with >10 years in practice were more likely to avoid oral CAI in patients with prior SA rash and/or urticaria than those with ≤10 (OR 2.27, *p* = 0.002). Respondents affiliated with non-glaucoma organizations were more likely to avoid oral CAI in patients with prior SA rash and/or urticaria than those affiliated with glaucoma organizations (*p* = 0.03). Providers without glaucoma training were more likely to avoid topical CAI in patients with prior SA rash and/or urticaria (*p* = 0.004) and anaphylaxis (*p* = 0.01) than glaucoma-trained providers.

**Conclusions:**

Despite no supporting evidence, a significant number of respondents would avoid CAI in patients with prior SA hypersensitivity. Respondents without glaucoma training, no affiliation with a glaucoma organization, and >10 years in practice are more likely to avoid CAI in patients with type I SA hypersensitivity. Providers should be informed of the low cross-reactivity risk between CAI and SA so more patients may benefit from these drugs.

## Introduction

**Fig. 1:**
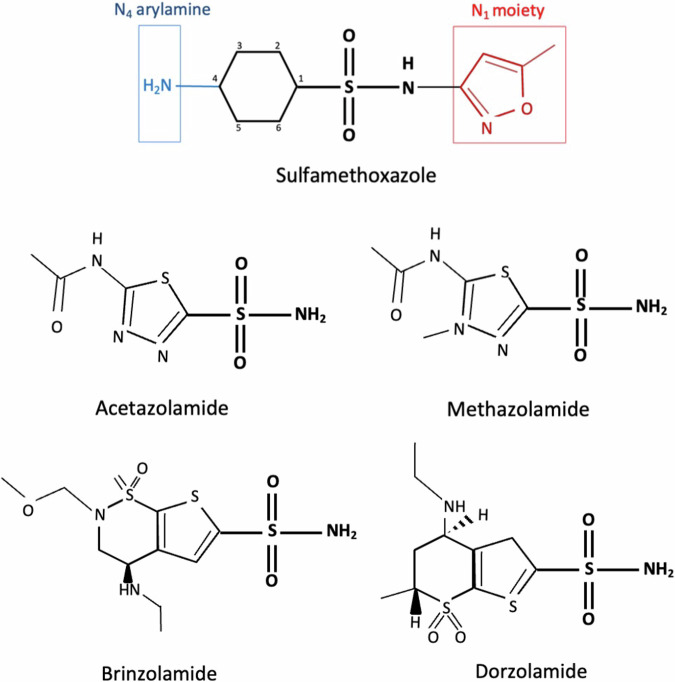
Sulfonamide antimicrobial molecular structure containing a sulfonamide group and N1 and N4 moieties, compared with commonly used ophthalmic CAIs.

Sulfonamide antimicrobials (SA) are one of the agents most frequently reported as causing drug hypersensitivity reactions [1, 2]. It has been estimated that 3–8% of patients exposed to these antimicrobials report an allergic drug reaction [1, 3, 4], although pharmacovigilance studies report that after complete investigation, true drug-hypersensitivity rate may be significantly overestimated [5, 6]. The term “sulfa allergy” was originally coined to refer to SA; however, it misleadingly became associated with any medications containing a sulfonamide group (-SO2NH2), which is not sufficient by itself to trigger hypersensitivity [7]. Two chemical structures in SA have been established as the antigenic determinants: a heterocyclic ring at the N_1_ position and, most importantly, an arylamine moiety at the N_4_ position of the benzene ring (Fig. 1) [8, 9]. While the former directly elicits a type I Ig E-mediated hypersensitivity, the latter undergoes biotransformation into metabolites responsible for the non-type I hypersensitivity by direct cytotoxicity, humoral, or cellular responses [10–13].

Carbonic anhydrase inhibitors (CAI) such as acetazolamide, methazolamide, dorzolamide, and brinzolamide are nonantimicrobial non-arylamine sulfonamides (NAS) used in ophthalmology mainly in the management of glaucoma and idiopathic intracranial hypertension. Because none of these ophthalmic CAI possess the N_1_ or N_4_ moieties [11, 14], a cross-reactivity between SA and CAI is unlikely. Observational studies have concluded that there is no compelling evidence to support cross-reactivity between SA and NAS [15–18]. Evidence rather points toward an idiosyncratic predisposition to a multiple drug hypersensitivity syndrome. Nonetheless, cautionary warnings for sulfonamide hypersensitivity or cross-reactivity remain in ophthalmic CAI manufacturers’ package inserts in several countries, including the United States [14, 19].

To our knowledge, eyecare providers’ practice patterns toward CAI prescription in SA-allergic patients have not been previously evaluated. We hypothesize that provider’s characteristics might affect CAI prescription in these patients. To test this hypothesis, we surveyed members of four US eyecare organizations to assess respondents’ attitudes toward CAI prescription in hypothetical SA-allergic patients with different types of hypersensitivity responses.

## Methods

The study protocol was approved by the Institutional Review Board of the University of Miami Miller School of Medicine. A self-administered electronic survey in English was designed using an online survey tool (www.surveymonkey.com, San Mateo, CA). The survey link and QR code were distributed to members subscribed to the mailing lists of the American Glaucoma Society (AGS), the American Association for Pediatric Ophthalmology and Strabismus (AAPOS), the Glaucoma Section of the American Academy of Optometry (GSAAO), and the Optometric Glaucoma Society (OGS). The survey was available from April to August 2021 and could be completed only once from each unique e-mail address.

The survey queried ophthalmologists and optometrists who had completed residency with or without specialty training about some specific characteristics as providers (profession, specialty, organizational affiliation, and years in training) and their CAI prescription habits in the past year. Participants who prescribed CAI on a regular basis (defined by the authors as more than twice a month) were eligible to continue to the clinical scenarios presented in Table 1.

**Table 1:** Response options listed in the survey for each of four clinical scenarios and frequencies chosen.

Clinical Scenarios		*N* (%)
Scenario 1. Assuming medical therapy only, for a patient with uncontrolled primary open angle glaucoma, and reported history of acute sulfonamide antibiotic rash, would you consider prescribing acetazolamide or methazolamide?		
No		130 (52)
Yes		120 (48)
Scenario 2. In an adult patient with primary open angle glaucoma, no comorbidities, and reported history of sulfonamide antibiotic anaphylactic reaction, would you consider prescribing brinzolamide or dorzolamide?		
No		123 (49.2)
Yes		127 (50.8)
Scenario 3. In a patient with history of sulfonamide antibiotic allergy, which of the following would prevent you from prescribing acetazolamide or methazolamide? You can choose all that apply^a^		
Acute Rash		107 (42.8)
Acute Urticaria		119 (47.6)
Anaphylaxis/Angioedema		226 (90.4)
Stevens-Johnson syndrome		228 (91.2)
None of the above^a^		12 (4.8)
Scenario 4. In a patient with history of sulfonamide antibiotic allergy, which of the following would prevent you from prescribing brinzolamide or dorzolamide? You can choose all that apply^a^		
Acute Rash		54 (21.6)
Acute Urticaria		60 (24.0)
Anaphylaxis/Angioedema		180 (72)
Stevens-Johnson syndrome		185 (74)
None of the above^a^		51(20.4)
Total		250 (100)

^a^Respondents could choose all alternatives that apply. However, they were instructed that if “None of the above” was chosen only “None of the above” will count as the response.

Associations among categorical variables were assessed using Pearson’s chi-squared test (X^2^) or Fischer’s exact test for binary variables with small, expected values. Forward stepwise logistic multivariable regression modeling was used to test whether provider’s characteristics were independently associated with respondent CAI avoidance in different hypersensitivity responses in hypothetical SA-allergic patients from the following variables: profession, self-reported specialty (glaucoma or non-glaucoma training), affiliation to an eyecare organization (glaucoma organization or non-glaucoma organization), and years in practice. At each step, variables were added based on *p*-values (*p* < 0.1) to set a limit on the total number of variables included in the final model. Effect estimates were summarized using odds ratios (OR) with 95% confidence intervals (95% CI) and *p*values. Statistical significance was set at *p* < 0.05. Statistical analyses were performed using the Stata v.17.0 statistical package (StataCorp LLC, College Station, TX, USA).

## Results

A total of 313 responses were received from 2532 active members of the AGS, AAPOS, GSAAO, and OGS (12.4% response rate). Sixty-three respondents were excluded from the final analysis: 19 due to survey incompleteness and 44 due to ineligibility (non-regular CAI prescription). Ultimately, 250 out of 266 (93.9%) of respondents eligible to continue to the clinical scenarios completed the survey. Most respondents were ophthalmologists (76.8%), reported glaucoma training (69.6%), were affiliated to an organization focused on glaucoma (82%) or had been practicing more than 10 years (64.2%), as described in Table 2. Nearly all respondents prescribed dorzolamide on a regular basis.

**Table 2:** Characteristics of respondents.

Provider’s Characteristics	*N*	%
Profession		
Ophthalmology	192	76.8
Optometry	58	23.2
Specialty^a^		
Glaucoma	174	69.6
Non- glaucoma	76	30.4
Organization^b^		
Glaucoma	205	82
Non-Glaucoma	45	18
Years in practice		
≤10 years	94	37.6
>10 years	156	62.4

CAI prescription^c^	N	%
Acetazolamide	182	72.8
Methazolamide	86	34.4
Dorzolamide	249	99.6
Brinzolamide	185	74

^a^Self-reported training in glaucoma or non-glaucoma specialty.

^b^Respondent affiliated or not with a glaucoma organization.

^**c**^Carbonic anhydrase inhibitor prescribed more than twice a month within the past year.

In the setting of a hypothetical patient with uncontrolled primary open-angle glaucoma and a reported history of SA-related acute rash (scenario 1), 130 (52%) respondents would avoid prescribing oral CAI (acetazolamide or methazolamide). Years in practice and organizational affiliation were significant in the univariable analysis, and these were the only variables that were independently associated in the multivariable model. Participants with more than 10 years in practice were more likely to avoid oral CAI in this scenario than those with 10 years or fewer (adjusted OR 2.32, 95% CI 1.36–3.93, *p* = 0.002). Participants who reported affiliation with a glaucoma organization were less likely to avoid oral CAI in this scenario compared to participants who reported no affiliation with a glaucoma organization (adjusted OR 0.46, 95% CI 0.23–0.92, *P* = 0.03). Similarly, 123 (49.2%) respondents would avoid prescribing a topical CAI (brinzolamide or dorzolamide) when a hypothetical patient with uncontrolled primary open-angle glaucoma with a history of SA anaphylaxis was presented (scenario 2). However, no significant difference in the provider’s characteristics was found in this scenario.

Given a hypothetical patient with a history of SA hypersensitivity in which no other clinical information was disclosed, 130 (52%) respondents would avoid prescribing oral CAI, if this was a mild cutaneous reaction, such as acute rash and/or urticaria (scenario 3). Years in practice was the only significant factor associated with a respondent’s decision to avoid oral CAI in this scenario. Participants with more than 10 years in practice are more likely to avoid oral CAI in patients with SA-related rash and/or urticaria than those with 10 years or fewer in practice (OR 2.27, 95% CI 1.35–3.84, *p* = 0.002) (Table 3). More than 90% of respondents would avoid oral CAI in patients with a history of SA-related anaphylaxis/angioedema or SA-related Stevens-Johnson syndrome, with no significant difference in the provider’s characteristics.

**Table 3:** Association between provider’s characteristics and avoidance of oral CAI prescription in a hypothetical patient with a history sulfonamide antibiotic allergy by hypersensitivity reaction (Scenario 3).

Characteristics	Acute Rash/Urticaria^a^		Anaphylaxis/Angioedema^b^			SJS^c^	
	Univariable OR (95% CI)	p-value	Univariable OR (95% CI)	p value	Univariable OR (95% CI)		p value
Profession							
OPH	Ref	—	Ref	—	Ref		—
OD	1.42 (0.78–2.57)	0.25	0.90 (0.34–2.37)	0.83	1.40 (0.45–4.30)		0.56
Specialty							
Non-glaucoma	Ref	—	Ref	—	Ref		—
Glaucoma	0.71 (0.41–1.22)	0.22	0.74 (0.28–1.95)	0.55	1.34 (0.54–3.35)		0.53
Organization							
Non-glaucoma	Ref	—	Ref	—	Ref		—
Glaucoma	0.84 (0.44–1.60)	0.60	0.90 (0.29–2.78)	0.86	2.33 (0.89–6.11)		0.08
Years in practice							
≤10 years	Ref	—	Ref	—	Ref		—
>10 years	**2.27 (1.35–3.84)**^d^	**0.002**^d^	0.99 (0.42–2.37)	0.99	1.43 (0.59–3.45)		0.43

*CAI* carbonic anhydrase inhibitors, *OPH* ophthalmologist, *OD* optometrist, *SJS* Stevens-Johnson syndrome, *Ref* reference category.

^a,b,c^Rate of avoidance of acetazolamide or methazolamide by HPS reaction: ^a^130/250 (52%), ^b^ 226/250 (90.4%), ^c^ 228/250 (91.2%).

^d^Final model: profession, organizational affiliation, and specialty do not affect statistical significance once the factor years in practice was adjusted for in the model. Bold face (statistically significant).

Concerning topical CAI prescription, 68 (27.2%) and 180 (72%) of respondents would avoid topical CAI in hypothetical patients with prior SA-related rash and/or urticaria and SA-related anaphylaxis/angioedema, respectively (scenario 4). Self-reported specialty and organizational affiliation were significantly associated with topical CAI avoidance in the former scenario in the univariable analysis, whereas specialty was the only significant factor for the latter. However, self-reported specialty was the only variable independently associated with topical CAI avoidance after forward stepwise multivariable regression modeling. Compared to those who reported no glaucoma training, respondents with glaucoma training were less likely to avoid topical CAI in patients with SA-related rash and/or urticaria (OR 0.43, *p* = 0.004) and SA-related anaphylaxis/angioedema (OR 0.42, *p* = 0.01), as depicted in Table 4. No significant association was found between any provider’s characteristics and avoidance of topical CAI in patients with history of SA-related Stevens-Johnson syndrome. We also did not find significant differences in CAI prescription attitudes between ophthalmologists and optometrists in any of the scenarios presented.

**Table 4:** Association between provider’s characteristics and avoidance of topical CAI prescription in a hypothetical patient with a history sulfonamide antibiotic allergy by hypersensitivity reaction (Scenario 4).

Characteristics	Acute Rash/Urticaria^a^		Anaphylaxis/Angioedema^b^		SJS ^c^	
	Univariable OR (95% CI)	*p* value	Univariable OR (95% CI)	*p* value	Univariable OR (95% CI)	*p* value
Profession						
OPH	Ref	—	Ref	—	Ref	-
OD	1.42 (0.75–2.69)	0.28	1.03 (0.53–1.98)	0.94	1.14 (0.57–2.25)	0.71
Specialty						
Non-glaucoma	Ref	—	Ref	—	Ref	—
Glaucoma	**0.43 (0.24–0.77**)^d^	**0.004**^d^	**0.42 (0.21–0.83)**^d^	**0.01**^d^	0.54 (0.28–1.06)	0.07
Organization						
Non-glaucoma	Ref	—	Ref	-	Ref	—
Glaucoma	**0.38 (0.19–0.75**)	**0.005**	0.59 (0.27–1.29)	0.19	0.90 (0.43–1.91)	0.79
Years in practice						
≤10 years	Ref	—	Ref	-	Ref	—
>10 years	1.81 (0.99–3.31)	0.06	0.68 (0.27–1.67)	0.40	1.62 (0.91–2.88)	0.10

*CAI* carbonic anhydrase inhibitors, *OPH* ophthalmologist, *OD* optometrist, *SJS* Stevens-Johnson syndrome, *Ref* reference category.

^a,b,c^ Rate of avoidance of dorzolamide or brinzolamide by HPS reaction: ^a^ 68/250 (27.2%), ^b^ 180/250 (72%), ^c^ 185/250 (74%).

^d^Final model through forward stepwise regression modeling: profession, affiliation to an eyecare organization and years in practice do not affect statistical significance once the specialty was adjusted for in the model. Bold face (statistically significant).

## Discussion

Reported SA allergy continues to complicate clinicians’ decision to prescribe CAI. Therapeutic decisions regarding CAI prescription for patients with a history of SA allergy can be challenging for both healthcare providers and patients. In this study, we asked eyecare specialist members of four US eyecare organizations to indicate which hypersensitivity reaction would prevent them from prescribing oral or topical CAI in the context of hypothetical patients with a history of SA allergy. We assumed that survey participants acknowledge that CAI are sulfonamide-containing drugs and that they have no reason to disguise their choice.

Overall, a significant proportion of respondents would avoid prescribing CAI in hypothetical SA-allergic patients. Avoidance rates increased with increasing hypersensitivity severity, and providers reported about a 20% higher reluctance to prescribe oral compared to topical CAI in equivalent scenarios. Approximately half of respondents would not prescribe oral CAI even if the SA hypersensitivity was an acute mild cutaneous reaction. Our analysis shows that, in a general setting, respondents with more than 10 years in practice are more than twice as likely than to those with 10 years or fewer in practice to avoid oral CAI in a hypothetical patient with prior mild type I SA hypersensitivity. In 2000, Balas and Boren estimated that it took approximately 15 years from the publication of landmark clinical trials to strongly affect clinical practice, and an average of 9.3 years to implement evidence from reviews, papers, and textbooks [20]. Accordingly, a plausible explanation of the lower avoidance rate among our younger respondents may be related to the fact that, although sulfonamide cross-reactivity retrospective landmark studies were reported within the last twenty years [15, 16, 18], several comprehensive review articles discussing the lack of evidence of cross-allergenicity between SA and NAS have been published within the past decade [12, 13, 21, 22].

When participants were presented a hypothetical glaucoma patient with a history of SA-related acute rash, both years in practice and organizational affiliation affected provider’s prescription of oral CAI in this scenario. Respondents who reported affiliation with a glaucoma organization were 54% less likely to avoid oral CAI compared to those who reported no affiliation with a glaucoma organization. It is possible that eyecare providers affiliated to glaucoma organizations were exposed to updated evidence concerning lack of cross-reactivity between SA and CAI through educational material or educational meetings provided/held by their organizations.

We also found that, when facing the decision to prescribe topical CAI in a hypothetical SA allergic patient without specifying the clinical indication, respondents who reported glaucoma training were nearly 60% less likely to avoid topical CAI in patients with SA-related type 1 hypersensitivity compared to those who reported no glaucoma training. We speculate that participants who reported glaucoma training may be aware of studies supporting the lack of cross-reactivity between CAI and SA.

Another finding in our study is the significant difference in the rate of topical CAI avoidance for hypothetical patients with a reported history of SA-related anaphylaxis under different contexts. When the respondents were presented a patient with uncontrolled primary open angle glaucoma and no comorbidity (scenario 2), 49.2% would avoid a topical CAI, compared to 72% when no other clinical data besides the history of SA-related anaphylaxis was disclosed to make the clinical decision (scenario 4). Prior studies reported that concomitant disease states and concurrent drug exposure might increase the risk for drug hypersensitivity by altering metabolic pathways and inducing a variation of immunologic responses [4, 5, 23]. Some participants may have perceived that a patient with no comorbidities, and thus unlikely to be under concurrent medications, would be at a lower risk of drug hypersensitivity response. Another possibility could be the perception of facing an urgent condition (uncontrolled primary open angle glaucoma), for which CAI are the drugs of choice.

In a similar study, Wall et al. surveyed licensed pharmacists to evaluate their attitudes toward filling and dispensing four systemic NAS in hypothetical patients with SA allergy [24]. Although the authors did not include a scenario with any ophthalmic CAI, between 40% to 85% of the respondents would not fill and dispense NAS in these patients, with the highest rate corresponding to zonisamide, an oral CAI sulfonamide.

The concern surrounding the potential cross-reactivity between SA and NAS appears to stem from a 1955 report of Moseley and Baroody on the use of oral acetazolamide as a diuretic in oedematous patients mostly due to cardiac aetiology. The authors postulated a potential cross-reaction in one patient with history of valvuloplasty due to mitral stenosis and self-reported “sulfa” allergy who developed respiratory distress, limbs oedema, and haemoglobinuria two days after re-administering 250 mg/day of acetazolamide for the treatment of congestive heart failure [25]. As later observed by Stock, these symptoms cited as an allergic reaction could be attributable to the patient’s worsening congestive heart failure status [26], considering acetazolamide’s weak diuretic action. Subsequently, only four more cases hypothesizing possible cross-reactivity with acetazolamide have been reported since this drug was approved in 1953 [27–30]. None of these cases clearly stated a history of SA hypersensitivity in the affected patient, and only one underwent a skin test which was positive for an unspecified sulfonamide solution [29]. In contrast, a recent series showed no allergic reactions after acetazolamide was administered in three patients with a self-reported history of severe SA rash [31]. Moreover, a single-masked study, found no cross-reactivity between SA and NAS, after skin test and controlled oral challenge test were performed in 28 patients with history of SA-induced fixed drug eruption [32].

To date, there is a limited number of observational studies that have evaluated cross-reactivity between SA and NAS, although no large-sample size research has specifically addressed cross-reactivity with CAI. The most relevant one is the large retrospective cohort reported by Strom et al. in 2003 who assessed the risk of allergic reactions within 30 days after receiving NAS among more than 20,000 patients. Their outcomes suggested that, although SA hypersensitivity increased the risk of subsequent hypersensitivity to NAS, a history of penicillin hypersensitivity was at least an equally strong risk factor. Moreover, patients with SA hypersensitivity subsequently exposed to penicillin showed a higher risk of hypersensitivity compared to those who received NAS [15]. The authors concluded that the initial association between SA and NAS appeared to be due to an individual predisposition to allergic reactions rather than to a sulfonamide cross-reactivity. A year later, Lee et al. found that 7% of patients who self-reported non-life-threatening SA hypersensitivity and received acetazolamide for intracranial hypertension experienced urticaria as the only allergic reaction after long-term follow-up [16]. Most recently, Guedes et al. evaluated adverse events in patients with self-reported hypersensitivity to any sulfonamide-containing drugs who later received topical antiglaucoma drugs (CAI and non-CAI). They concluded that it may be safe to use topical CAI in patients with self-reported sulfa allergy because the rate of systemic reactions was not significantly different among topical medication classes [18]. Like Strom, the authors suggested that patients with a history of any medication allergies may be more likely to develop allergic reactions to other, unrelated drug classes.

There are several barriers and factors that could explain most clinicians’ attitudes and behavior toward CAI prescription in SA-allergic patients, despite pharmacological and clinical evidence do not support cross-reactivity between SA and NAS. First is the use of the term “sulfa allergy” to refer to allergic reactions related to all sulfonamide-containing drugs despite evidence that the N_1_ and N_4_ moieties—neither of which is present in ophthalmic CAIs—are responsible for the SA allergic response. A second factor could be the lack of randomized clinical trials or large prospective studies that could validate the lack of cross-reactivity suggested by the retrospective ones. Nevertheless, we believe that the most important factor that contributes to the concern among patients and clinicians is the manufacturers’ package inserts for ophthalmic CAIs that contain warnings regarding sulfonamides hypersensitivity or cross-reactivity [19]. These warnings could be a deterrent for clinicians out of fear of a possible severe allergic reaction in their patients and medico-legal liability from disregarding FDA-approved cautionary recommendations, even though these would not be aligned with current available evidence.

Our study had several limitations. Our response rate is modest and may not reflect the true prescribing patterns among eyecare providers in general. Although ophthalmologists constituted the larger number of respondents in our study, only 10.6% of active members in the two ophthalmology organizations responded to the survey compared to 26.2% of the optometric organizations’ members. It is possible that the topic addressed was not of high priority for invitees who do not manage glaucoma and/or prescribe CAI. Selection-bias may have also been introduced by excluding participants who prescribed CAI twice a month or less and by not including other non-glaucoma organizations.

Our findings suggest that, despite the lack of scientific evidence supporting cross-reactivity between SA and CAI, a significant number of our respondents would avoid these drugs in patients with a history of SA hypersensitivity, potentially withholding sight-saving therapy from a significant portion of glaucoma patients. In general, eyecare providers who reported no affiliation with a glaucoma organization, no glaucoma training and/or more than 10 years in practice were more likely to avoid CAI prescription in patients with history of SA type I hypersensitivity than glaucoma organization affiliates, glaucoma-trained and/or younger colleagues. CAI are among the most effective and widely available drugs used in several ophthalmologic conditions. Hence, active educational efforts may increase awareness of the low risk of cross-reactivity between CAI and SA, which may allow more patients to benefit from these drugs. Further research could also explore the determinants for eyecare specialists’ implementation of evidence-based medicine into their clinical practice regarding the safe use of CAI in these patients.

## Summary

### What was known before

- Carbonic anhydrase inhibitors are often avoided in patients with sulfonamide antibiotics due to concern for a potential allergy, even without compelling evidence supporting true cross-reactivity.
- Eyecare provider practice patterns in these situations has not been previously evaluated.

### What this study adds

- Despite no compelling evidence for cross-reactivity between sulfonamide antibiotics and carbonic anhydrase inhibitors, a significant number of eyecare providers would avoid these drugs in patients with a history of sulfonamide antibiotic allergy.

## Data Availability

The datasets used and/or analysed during the current study are available from the corresponding author upon reasonable request.
